# *Krameria lappacea* root extract’s anticoccidial properties and coordinated control of CD4 T cells for IL-10 production and antioxidant monitoring

**DOI:** 10.3389/fimmu.2024.1404297

**Published:** 2024-05-01

**Authors:** Rewaida Abdel-Gaber, Ghada Alamari, Mohamed A. Dkhil, Andreas Meryk, Esam M. Al-Shaebi, Saleh Al-Quraishy

**Affiliations:** ^1^ Department of Zoology, College of Science, King Saud University, Riyadh, Saudi Arabia; ^2^ Department of Zoology and Entomology, Faculty of Science, Helwan University, Cairo, Egypt; ^3^ Applied Science Research Center, Applied Science Private University, Amman, Jordan; ^4^ Department of Pediatrics, Medical University of Innsbruck, Innsbruck, Austria

**Keywords:** *Eimeria papillata*, oxidative damage, *Krameria lappacea*, CD4 T cells, NFkB cells, IL-10

## Abstract

**Introduction:**

Recently, the use of botanicals as an alternative to coccidiostats has been an appealing approach for controlling coccidiosis. Therefore, this study was conducted to evaluate the potential role of aqueous methanolic extract (200 mg/kg) of *Krameria lappacea* (roots) (KLRE) against infection induced by *Eimeria papillata*.

**Methods:**

A total of 25 male C57BL/6 mice were divided into five groups (I, II, III, IV, and V). On 1^st^ day of the experiment, all groups except groups I (control) and II (non-infected-treated group with KLRE), were inoculated orally with 10^3^ sporulated *E. papillata* oocysts. On the day of infection, group IV was treated with KLRE. Group V served as an infected-treated group and was treated with amprolium (coccidiostat).

**Results:**

Treatment with extract and coccidiostat was continued for five consecutive days. While not reaching the efficacy level of the reference drug (amprolium), KLRE exhibited notable anticoccidial activity as assessed by key criteria, including oocyst suppression rate, total parasitic stages, and maintenance of nutrient homeostasis. The presence of phenolic and flavonoid compounds in KLRE is thought to be responsible for its positive effects. The *Eimeria* infection increased the oxidative damage in the jejunum. KLRE treatment significantly increased the activity of catalase and superoxide dismutase. On the contrary, KLRE decreased the level of malondialdehyde and nitric oxide. Moreover, KLRE treatment decreased macrophage infiltration in the mice jejunal tissue, as well as the extent of CD4 T cells and NFkB. *E. papillata* caused a state of systemic inflammatory response as revealed by the upregulation of inducible nitric oxide synthase (*iNOs*)-mRNA. Upon treatment with KLRE, the activity of *iNOs* was reduced from 3.63 to 1.46 fold. Moreover, KLRE was able to downregulate the expression of pro-inflammatory cytokines interferon-γ, nuclear factor kappa B, and interleukin-10 -mRNA by 1.63, 1.64, and 1.38 fold, respectively. Moreover, KLRE showed a significant reduction in the expression of IL-10 protein level from 104.27 ± 8.41 pg/ml to 62.18 ± 3.63 pg/ml.

**Conclusion:**

Collectively, *K. lappacea* is a promising herbal medicine that could ameliorate the oxidative stress and inflammation of jejunum, induced by *E. papillata* infection in mice.

## Introduction

The host immune response to the parasitic species is complex. Cell-mediated immunity driven by T lymphocytes, macrophages, and other effector cells plays a major role in host protection against coccidiosis (1). Upon activation by invading the coccidian *Eimeria* parasite, CD4 T cells can differentiate into various types of helper T cells that are responsible for regulating host immune responses by secreting cytokines and proinflammatory molecules (2). *Eimeria* species are highly species and site-specific within the host. *Eimeria papillata* is a significant cause of murine intestinal disease “coccidiosis” in mice jejunum. *E. papillata* is an intracellular obligate protozoan parasite having a complex life cycle of 5 days, comprising two developmental stages of exogenous (involving sporogony) and endogenous (involving schizogony and gametogony) within the host. Massive colonization of coccidia causes significant epithelial damage (3). The host may consequently suffer from diarrhea, malabsorption, inadequate weight gain, and overall poor performance (4). Also, coccidiosis leads to immune dysfunction and increases susceptibility to secondary bacterial infections, as it disrupts the balance of intestinal microflora (5).

Effective management of coccidiosis relies on vaccination and the use of chemoprophylaxis. However, the widespread and excessive use of synthetic coccidiostats has led to the development of drug resistance among various parasites (5–10). Consequently, alternative strategies for coccidiosis control have rapidly emerged and developed in response to these challenges. These strategies include the use of live anticoccidial vaccines, immunomodulators, prebiotics, and natural herbs (11). The utilization of various plants and their constituent parts for the control and treatment of *E. papillata* infection in mice has been documented. These plants have demonstrated therapeutic effects against coccidiosis, reducing mortality rate, oocyst numbers, and diarrhea, while also improving lesion scoring and production performance (12).

*Krameria lappacea*, commonly known as rhatany, belongs to the Krameriaceae family. It is renowned for its health-promoting characteristics attributed to its abundance in tannins, lignan derivatives, oligomeric proanthocyanidins, and benzofuran derivatives. Earlier investigators have confirmed their role as antioxidant (13), photoprotective (13), anti-inflammatory (14), antidiabetic (15), vasoprotective (16), anticancer (17, 18), and antimicrobial (19–22) activities. Moreover, its constituents are employed to alleviate diverse illnesses, among these, infections of the respiratory airways and gastrointestinal disorders (23).

Due to all the previously mentioned properties, this study was designed to investigate the anticoccidial and the antioxidant activity of *Krameria lappacea* roots extract (KLRE), as well as its role in the modulation of the expression of the inflammatory cytokines’ mRNA in the male C57BL/6 mice jejunum infected with the protozoan, *Eimeria papillata*.

## Materials and methods

### Methanolic extract preparation

The roots of *Krameria lappacea*, also known as rhatany, were obtained from a local market in Riyadh, Saudi Arabia A taxonomist from the Herbarium, College of Science (King Saud University), certified the plant identity with voucher number KSU-22958. The method outlined by Manikandan et al. (24) was employed to create a 70% methanolic extract from the roots of *K. lappacea*, referred to as KLRE. The crude extract was lyophilized and kept at -20°C.

### Total phenolics and flavonoids in KLRE

The total phenolic content of KLRE was measured using the Folin-Ciocalteu procedure, as reported by Abdel Moneim (25). Also, the total flavonoid content of KLRE was estimated using the aluminum chloride colorimetric method published by Abdel Moneim (25). Absorbance was measured with the Spectra MAX 190 (SoftMax^®^ Pro v.6.3.1). The values for phenolics and flavonoids are expressed as mg gallic acid/gram and mg quercetin/gram, respectively.

### The radical scavenging activity of 2,2-diphenyl-1-picrylhydrazyl

KLRE was found to be active in scavenging DPPH radicals. Initially, a fresh 0.08 mM DPPH radical solution was prepared in methanol. Then, 950 ml of the solution was combined with 50 ml of KLRE and incubated at 25°C for 5 minutes in the dark. The absorbance was measured at 515 nm using the Spectra MAX 190 (SoftMax^®^ Pro v.6.3.1). Akillioglu and Karakaya (26) measure antioxidant activity as the percentage reduction of DPPH radicals.

### Parasite passage

*E. papillata* was used as a model coccidian murine parasite. To propagate oocysts, five laboratory mice (*Mus musculus*) were infected with 10^3^ sporulated oocysts through oral gavage. Feces were collected at 5 days post-infection (p.i.), and oocysts were isolated using the floatation technique (27). Part of these oocysts could sporulate in 2.5% (*w/v*) potassium dichromate (K_2_Cr_2_O_7_) for three days before being washed in a phosphate buffer solution (Sigma Aldrich, Taufkirchen, Germany) and used in the *in vivo* study.

### *In vivo* infection and experimental design

The animal facility of King Saud University (Riyadh, Saudi Arabia) provided twenty-five male C57BL/6 mice (10-12 weeks old, weighing 20-25 g). All mice were bred under pathogen-free conditions and allowed food and water *ad libitum*. Animals were housed in plastic cages under temperature-controlled conditions with a 12-hour light/dark cycle. Animals had been acclimated for one week before the start of the experiment. Mice were divided into five groups of five mice per group, as follows: G-I, Non-infected, non-treated (negative control), G-II, Non-infected, treated group with 200 mg/kg KLRE (28), G-III, Infected, non-treated (positive control), G-IV, Infected and treated group with 200 mg/kg KLRE (28), and G-V: Infected and treated group with 120 mg/kg Amprolium (29) for 5 days. All groups (except 1 and 2) were orally infected with 10^3^ sporulated *E. papillata* oocysts in 100 µl physiological saline (30).

### Oocyst suppression

On day 5 p.i., fresh fecal pellets were collected from infected untreated and treated groups. The suppression of oocyst shedding was estimated using the formula of 100 - (oocysts output in the treated group/oocysts output in the infected group) × 100.

### Collection of jejunal samples

Mice were killed by CO_2_ asphyxiation on the 5^th^ day p.i., and mice’s jejuna were isolated and cut into small pieces for the following: (a) Neutral buffered formalin was utilized for histological analysis. (b) In small tubes maintained at -80°C to investigate the oxidative status and protein expression (c) RNA later^®^ (Invitrogen, Carlsbad, CA) was utilized for molecular analysis and kept at -80°C.

### Histological examination and parasitic score

The jejuna were fixed in 10% neutral buffered formalin (NBF) for 24 hr, dehydrated, and embedded in paraffin wax, and then cut into 5 µm thick sections as per Adam and Caihak (31) method. Sections of jejuna were stained with hematoxylin-eosin (H&E) to detect parasite stages in both infected and infected-treated groups. The slides were examined and photographed using an Olympus B×61 microscope (Tokyo, Japan), and parasitic stages were counted on ten well-oriented villous-crypt units (VCU).

### Histochemical examination

Other jejunal sections were stained using Hotchkiss (32) periodic acid-Schiff’s procedure for total carbohydrates and Mazia et al. (33) mercuric bromophenol blue method for total proteins. The slides were photographed using an Olympus B×61 microscope (Tokyo, Japan).

### Immunohistochemistry detection of CD4

Sections (5µm thick) were picked onto Superfrost^®^ glass slides (Thermo Scientific) and air-dried. Sections were deparaffinized using xylene and rehydrated through a serial grade of ethanol. Antigen retrieval was accomplished by steaming the slides in phosphate-buffered saline (PBS/pH=7.4) at various temperatures. To reduce non-specific background staining caused by endogenous peroxidase, H_2_O_2_ (3%) methanol solution was used. For immunostaining, the horseradish peroxidase amplified system, CD4 and NF-kB (nuclear factor kappa B) antibodies were used (Thermo Fisher Science, Waltham, MA, USA). Three components were used in this system: the primary antibody specific for the antigen to be localized, the secondary antibody capable of binding both primary antibodies and the horseradish peroxidase enzyme. Finally, the substrate/chromogen reagent diaminobenzidine (DAB) was used for reaction visualization. The number of immuno-histochemical-positive cells was presented as the mean number of brown cells per ten well-oriented villous-crypt units (VCU) and identified using an Olympus B×61 microscope (Tokyo, Japan).

### Biochemical analysis

To get 10% (w/v) jejunal homogenate, parts of jejunum were weighed, homogenized in an ice-cold medium with 50 mM Tris-HCl and 300 mM sucrose, and centrifuged for 10 min at 500×g at 4°C. The homogenate was then stored at -20°C until use (34). Oxidative stress markers were detected in the supernatant of jejunal homogenate. The appropriate chemical kits (Bio-Diagnostic kits, Bio-Diagnostic Co., Egypt) were used to determine Catalase (CAT), Nitric Oxide (NO), Malondialdehyde (MDA), and Superoxide Dismutase (SOD). Absorbance was measured with Spectra MAX 190 supported by software SoftMax^®^ Pro v.6.3.1.

### RNA extraction and qRT-PCR

Total RNA was extracted from jejunal tissues using Trizol (Invitrogen, USA). RNA samples were processed with DNase (Applied Biosystems, Darmstadt, Germany) for at least 1 hour before being transformed into cDNA according to the manufacturer’s instructions using a reverse transcription kit (Qiagen, Hilden, Germany). qRT-PCR was carried out using an ABI Prism 7500HT sequence detection system (Applied Biosystems, Darmstadt, Germany) with Qiagen’s SYBR green PCR master mix (Hilden, Germany). The mRNA genes for interferon- γ (IFN- γ), inducible nitric oxide synthase (iNOs), interleukin-10 (IL-10), NF-kB, as well as beta-actin (β-actin) as a housekeeping control were studied using SYBR green (Hilden, Germany). All primer assays for qRT-PCR were obtained from Qiagen (Hildan, Germany). Real-time qPCR amplification and analysis were carried out using the Bio-Rad IMark Microplate Reader SW 1.04.02.E. The Ct method (2^−ΔΔCT^) described by Livak and Schmittgen (35) was used to analyze differences in gene expression.

### Sandwich enzyme-linked immunosorbent assay for IL-10

Using a mouse IL-10 ELISA kit (IOK-05-P361, Creative Biolabs, USA) and following the protocol instructions, optical densities (OD) of outcomes from the jejunal samples were measured using the Bio-Rad IMark Microplate Reader SW 1.04.02.E. Based on a standard curve, OD values were converted to concentrations and presented as pg/ml.

### Statistical analysis

Values were presented as mean ± standard deviation (SD). A one-way analysis of variance (ANOVA) with Duncan’s test was performed to compare the group means, with *p*-value ≤ 0.05 indicating statistical significance. SPSS version 18 for Windows (SPSS Inc., Chicago, Illinois, USA) was used for the analysis.

## Results

**Figure 1:**
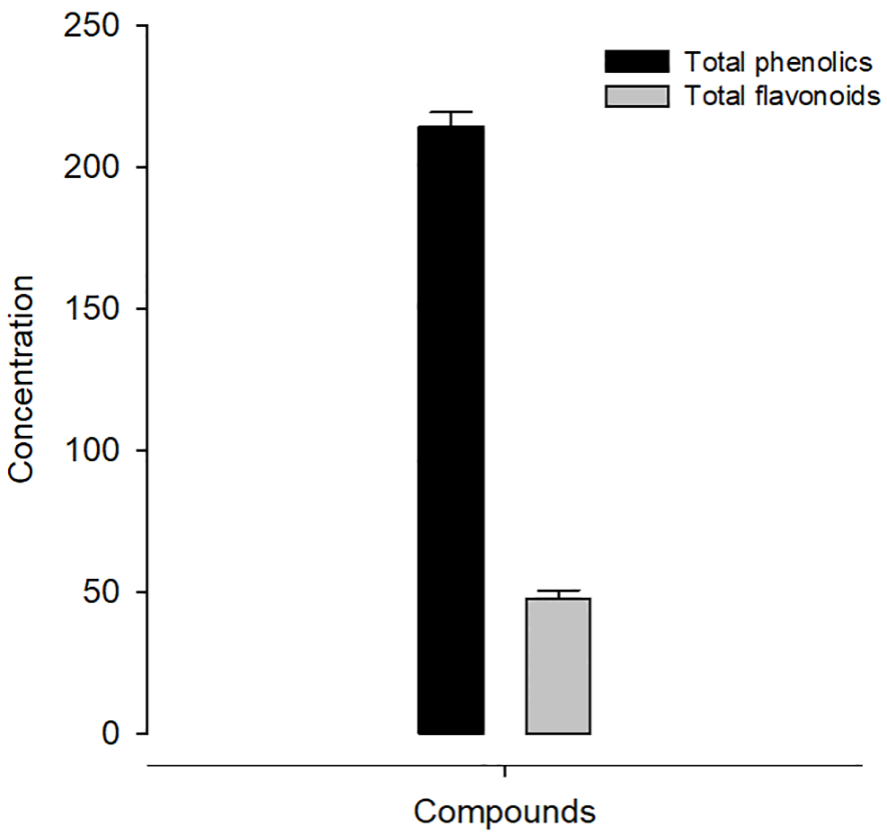
Concentration of phenolics (mg) and flavonoids (mg) in KLRE.

Using the Folin–Ciocalteu technique, KLRE had a total phenolic content of 214.30 ± 5.19 mg gallic acid/gram dry weight (**Figure 1**). Furthermore, the total flavonoid in KLRE measured using the aluminum chloride colorimetric method was 47.40 ± 3.20 mg quercetin/gram dry weight (**Figure 1**). KLRE’s antioxidant activity was measured using the 2,2-diphenyl-1-picrylhydrazyl (DPPH) method to measure free radical scavenging activity. **Table 1** showed that KLRE had maximum DPPH (90.69%) at 250 µg/ml, while the lowest scavenging percentage (13.20%) occurred at 15.625 μg/ml.

**Table 1:** DPPH radical scavenging activity (%) of roots extract for *Krameria lappacea* (KLRE).

Concentrations (µg/mL)	DPPH Radical Scavenging Activity (%)
15.625	13.20 ± 0.90
31.250	52.93 ± 0.78
63.500	73.30 ± 0.67
125.00	85.39 ± 0.49
250.00	90.69 ± 0.35
500.00	87.25 ± 0.69

**Figure 2:**
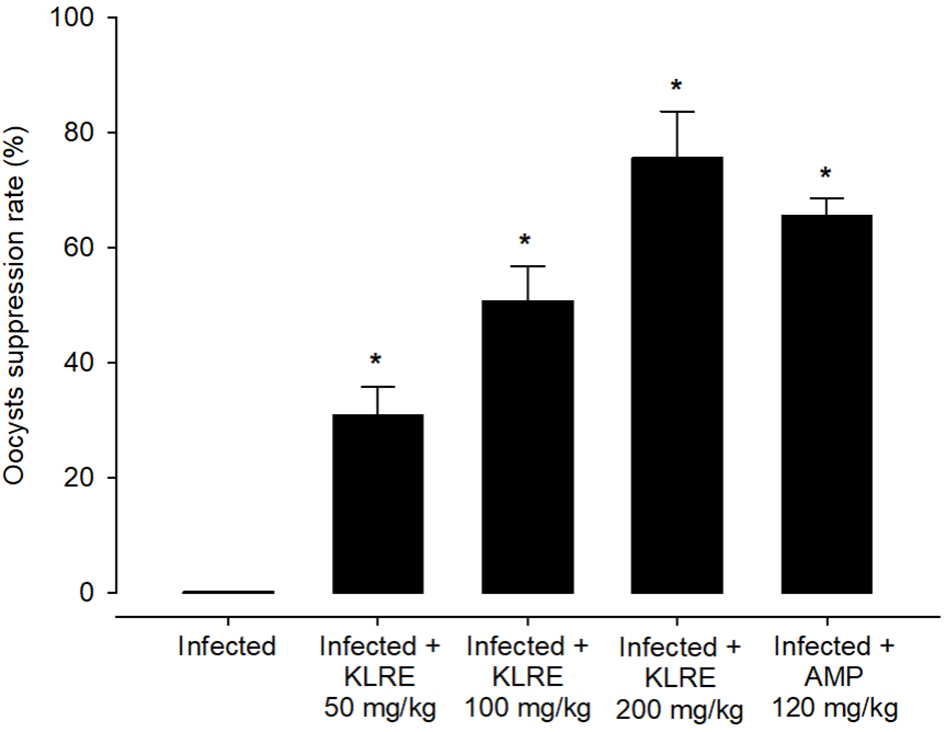
Suppression of *E. papillata* oocysts in infected and infected-treated mice. Significance At p ≤ 0.05 against the infected group (*).

**Figure 3:**
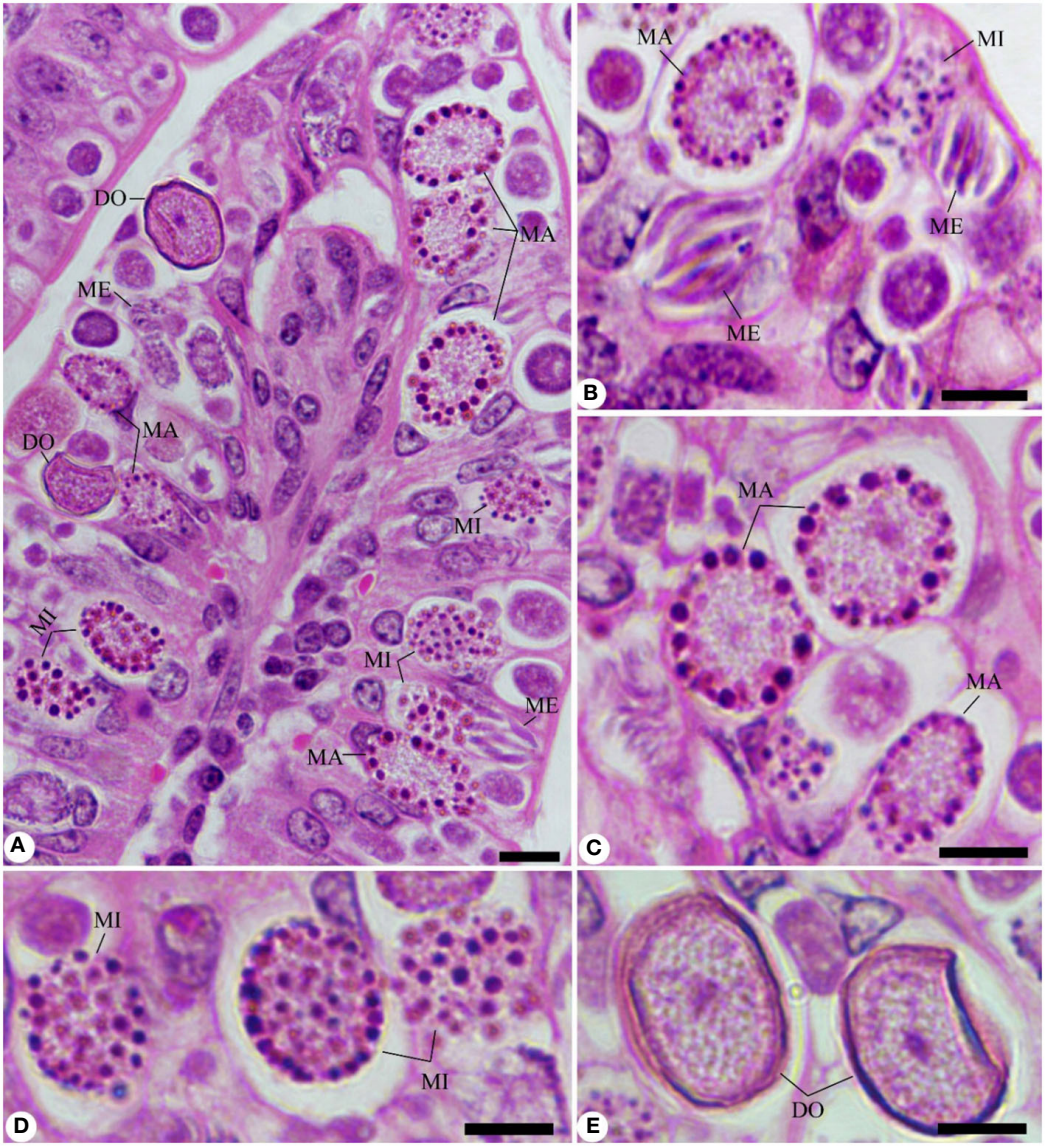
Sections stained with hematoxylin and eosin (H&E) for the infected jejunum with *Eimeria papillata* on day 5 p.i. showing different developmental stages. **(A, B)** Infected jejunum with different Eimeria stages. **(C)** Macrogamonts. **(D)** Microgamonts. **(E)** Developing oocysts. Note: ME, meronts; MA, macrogamont; MI, macrogamont; DO, developing oocyst. Scale bar = 10 µm.

**Figure 4:**
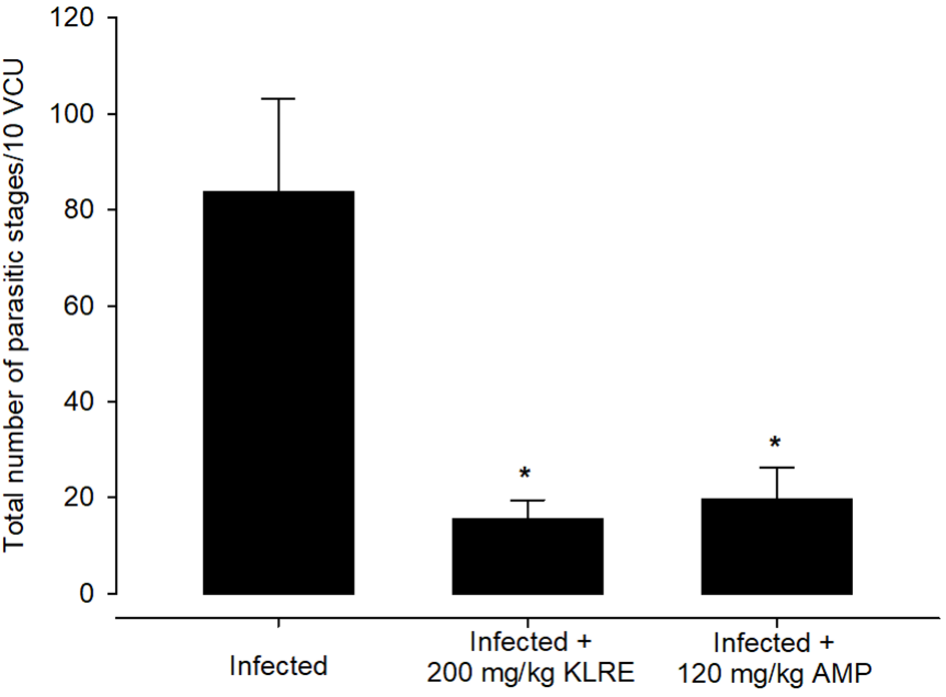
Treatment with 200 mg/kg KLRE and 120 mg/kg AMP induced changes in the total number of parasitic stages of *Eimeria papillata* in jejunum tissue per 10 VCU on day 5 p.i. All values are means ± SD. * significance (P ≤ 0.05) between the infected group and treated group.

Experimental *E. papillata* infection in infected and infected-treated mice groups was established as showed oocyst discharge in fecal pellets with a maximal level on the 5^th^ day of infection. After treatment with KLRE, the output of *Eimeria* oocysts was inhibited by 75.71%, which was higher than the reference drug (65.65%) (**Figure 2**). Light microscopic examinations of hematoxylin/eosin stained sections indicated the presence of developmental *Eimeria* stages in the epithelial cells of the jejunal mouse tissue (**Figure 3**). KLRE treatment significantly reduced the total number of intracellular murine *Eimeria* stages, from 83.75 ± 19.36 stages/10 VCU in the infected group to 15.43 ± 4.07 stages/10 VCU in mice infected with *E. papillata* and treated with KLRE, and 19.73 ± 6.51 stages/10 VCU in mice treated with amprolium (**Figure 4**).

**Figure 5:**
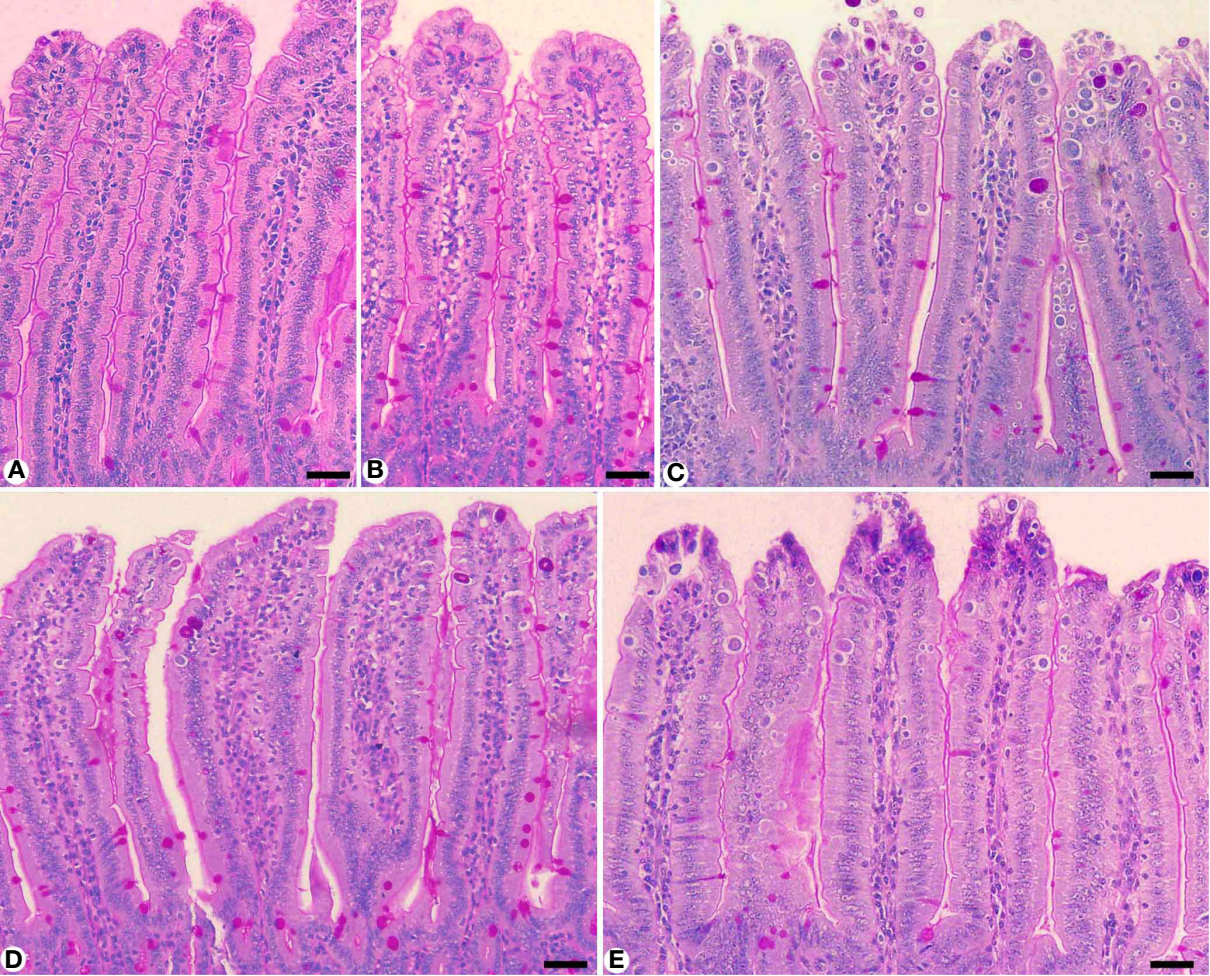
Carbohydrate content in jejunum sections stained with periodic acid Schiff’s (PAS) method. **(A)** control non-infected jejunum with normal content. **(B)** non-infected-treated group with 200 mg/kg KLRE. **(C)** *E*. *papillata* infected jejunum with depletion in their carbohydrate content. **(D, E)** infected treated mice (200 mg/kg KLRE and 120 mg/kg AMP, respectively) with improvement in their level. Scale bar = 100µm.

**Figure 6:**
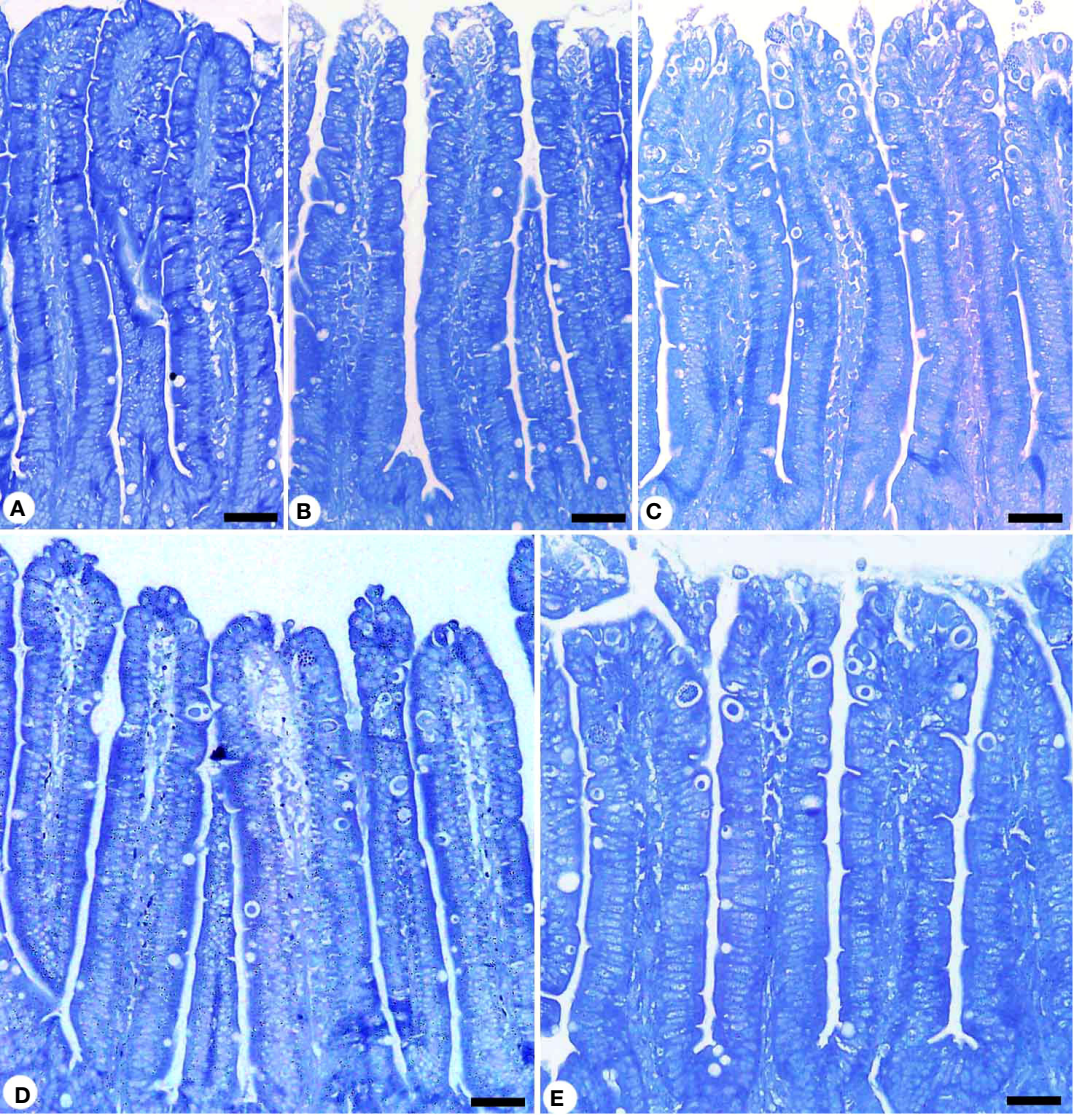
Protein content in jejunum sections stained with mercuric bromophenol blue method. **(A)** control non-infected jejunum with normal content. **(B)** non-infected-treated group with 200 mg/kg KLRE. **(C)** *E*. *papillata* infected jejunum with depletion in their protein content. **(D, C)** infected treated mice (200 mg/kg KLRE and 120 mg/kg AMP, respectively) with improvement in their level. Scale bar = 100µm.

Infection with *E. papillata* significantly altered the nutritional contents of the jejunal tissues. This was evidenced by the decrease in total carbohydrate content in the jejunal tissue for the infected group compared to the control one (**Figure 5**). KLRE treatment resulted in a significant change in carbohydrates compared to the infected group. Furthermore, *E. papillata* infection reduced jejunal protein content in contrast to the control group (**Figure 6**). KLRE treatment restored jejunal proteins to levels comparable to the infected group.

**Figure 7:**
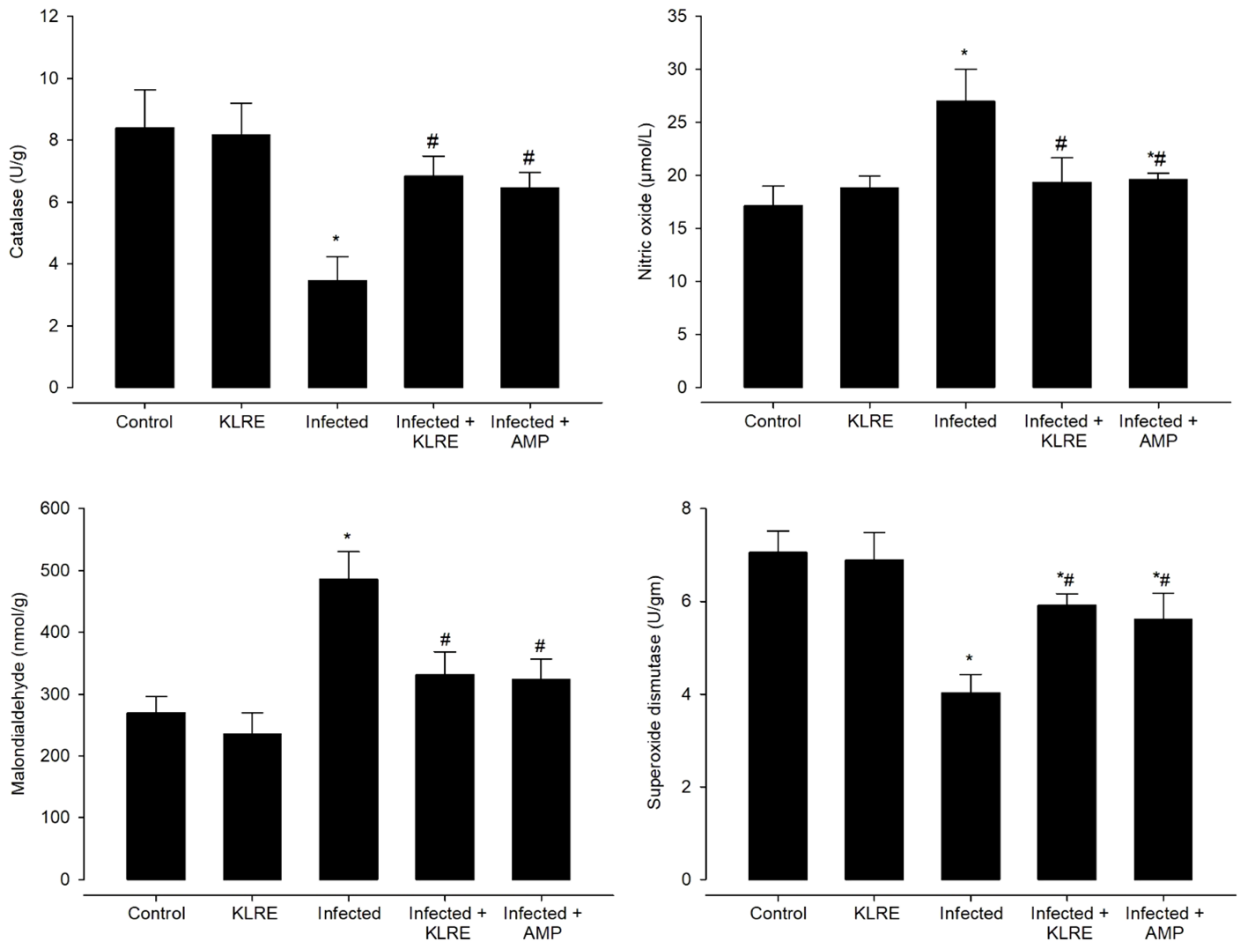
Effect of KLRE on the level of catalase, nitric oxide, malondialdehyde, and superoxide dismutase in mice infected with *Eimeria papillata*. ^*^ significant change concerning the control group, ^#^ significance change concerning the infected group.

CAT levels declined significantly from 8.40 ± 1.23 U/g in the non-infected group to 3.45 ± 0.78 U/g in the infected group. Mice treated with KLRE had higher levels of CAT (6.82 ± 0.66 U/g) compared to mice treated with the reference drug (6.44 ± 0.50 U/g) (**Figure 7**). Concerning NO production, *E. papillata* infection significantly increased jejunal NO, a free radical, to 26.99 ± 3.02 µmol/L compared to 17.13 ± 1.84 µmol/L in the control group. This marker contributes to the termination of lipid peroxidation reactions. Treatment with KLRE and amprolium resulted in considerable reductions in NO levels to 19.36 ± 2.29 and 19.59 ± 0.62 µmol/L, respectively (**Figure 7**). The infected group had a significantly higher level of malondialdehyde (MDA), a byproduct of polyunsaturated fatty acids peroxidation, compared to the non-infected group (269.71 ± 26.66 nmol/g *vs.* 485.62 ± 44.63 nmol/g). Mice treated with KLRE and amprolium significant decreases in MDA levels to 331.36 ± 36.93 and 323.03 ± 33.74 nmol/g, respectively (**Figure 7**). Furthermore, the activity of SOD reduced significantly from 7.05 ± 0.46 U/gm in the non-infected group to 4.03 ± 0.39 U/gm in the infected group. Mice treated with KLRE and amprolium showed significantly higher levels of SOD (5.91 ± 0.24 and 5.60 ± 0.56 U/gm, respectively) (**Figure 7**).

**Figure 8:**
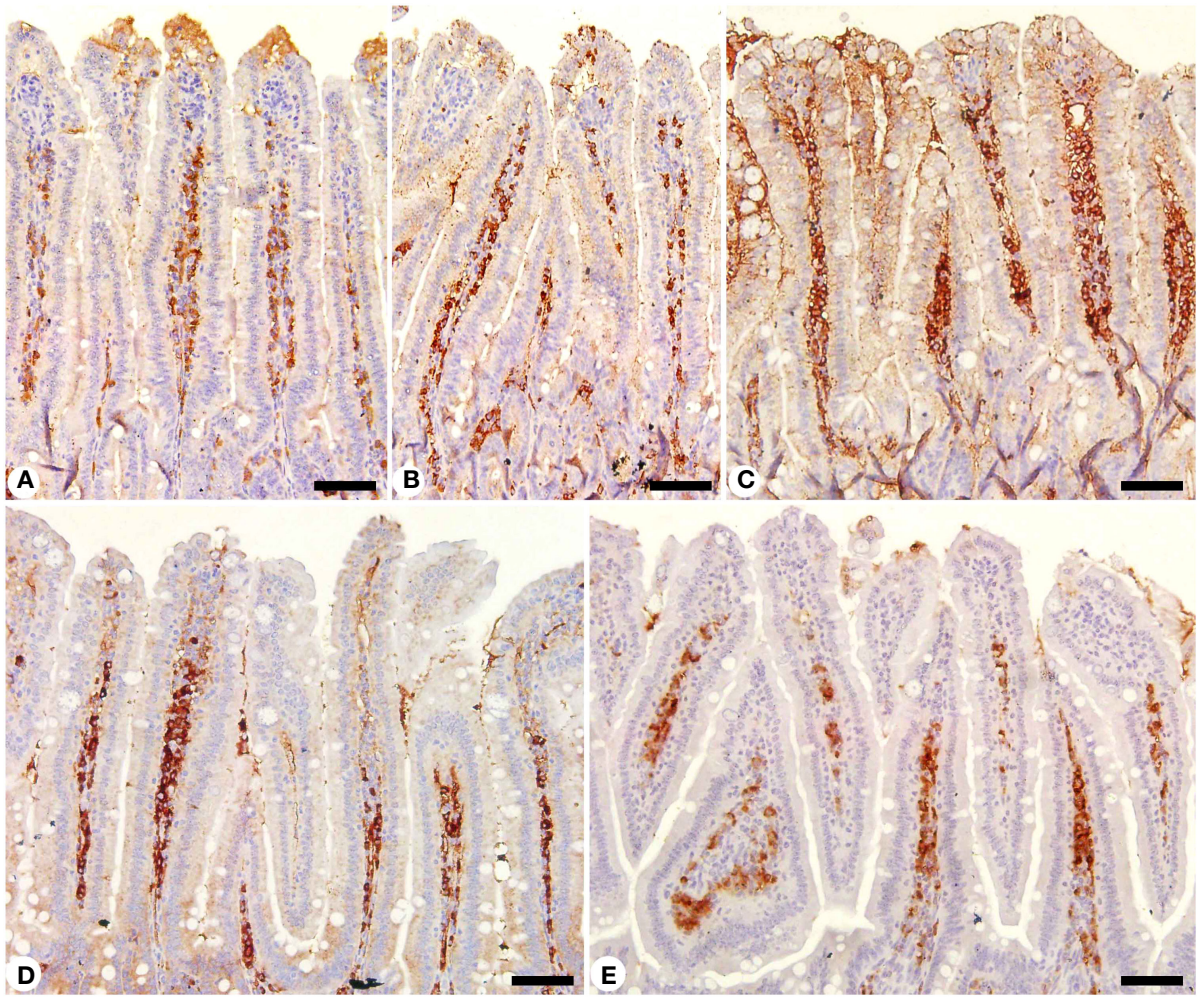
Immunohistochemical localization of CD4 in the jejuna of mice. **(A)** control non-infected jejunum. **(B)** non-infected-treated group with 200 mg/kg KLRE. **(C)** *E. papillata* infected jejunum with an increased number of CD4-positive cells. **(D, E)** infected treated mouse (200 mg/kg KLRE and 120 mg/kg AMP, respectively) with a decreased number of CD4-positive cells. Scale bar = 50µm.

**Figure 9:**
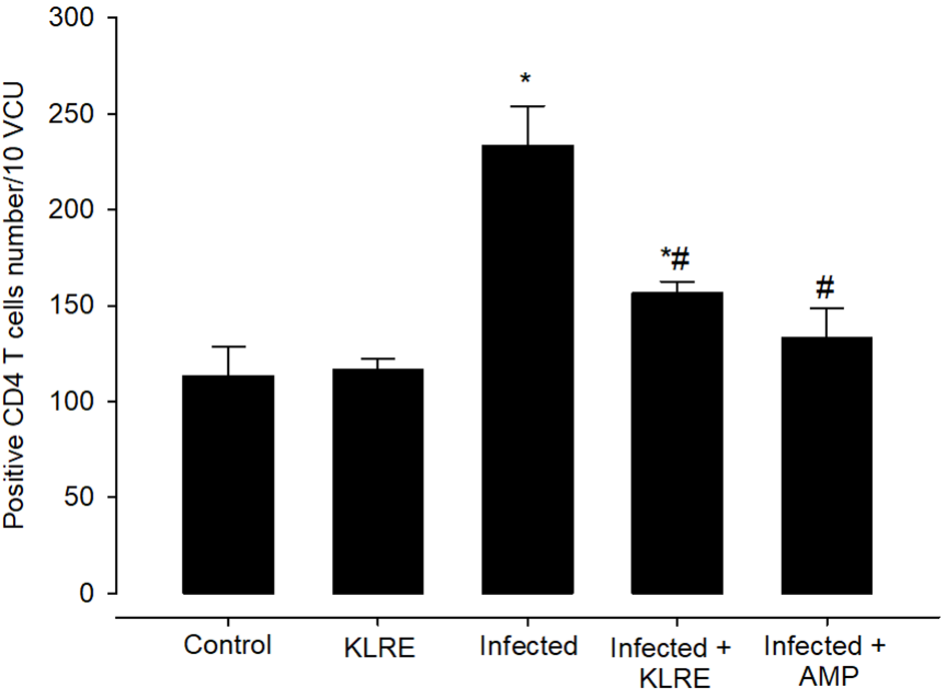
Positive CD4 T cells number in mice infected with *Eimeria papillata* and for infected treated groups with 200 mg/kg KLRE and 120 mg/kg AMP. ^*^ significant change concerning the control group, ^#^ significance change concerning the infected group.

Jejunal sections from experimental groups were stained for CD4 expression, which is thought to play an important role in the management of primary *E. papillata* infections (**Figure 8**). The *Eimeria* infection increased infiltration of positive immunohistochemical staining CD4 T cells into the infected mice jejunum, with the infected group having 233.33 ± 20.81 cells/10 VCU more positive cells than the control group, which had 113.33 ± 15.27 cells/10 VCU (**Figure 9**). CD4 T lymphocytes in murine coccidiosis may produce soluble cytokines such as IFN-γ and IL-10. Treatment resulted in a significantly lower CD4 expression (156.66 ± 5.77 cells/10 VCU) in the infected-treated KLRE group and (133.33 ± 15.27 cells/10 VCU) in the infected-treated amprolium group compared to the infected group (**Figures 8**, **9**).

**Figure 10:**
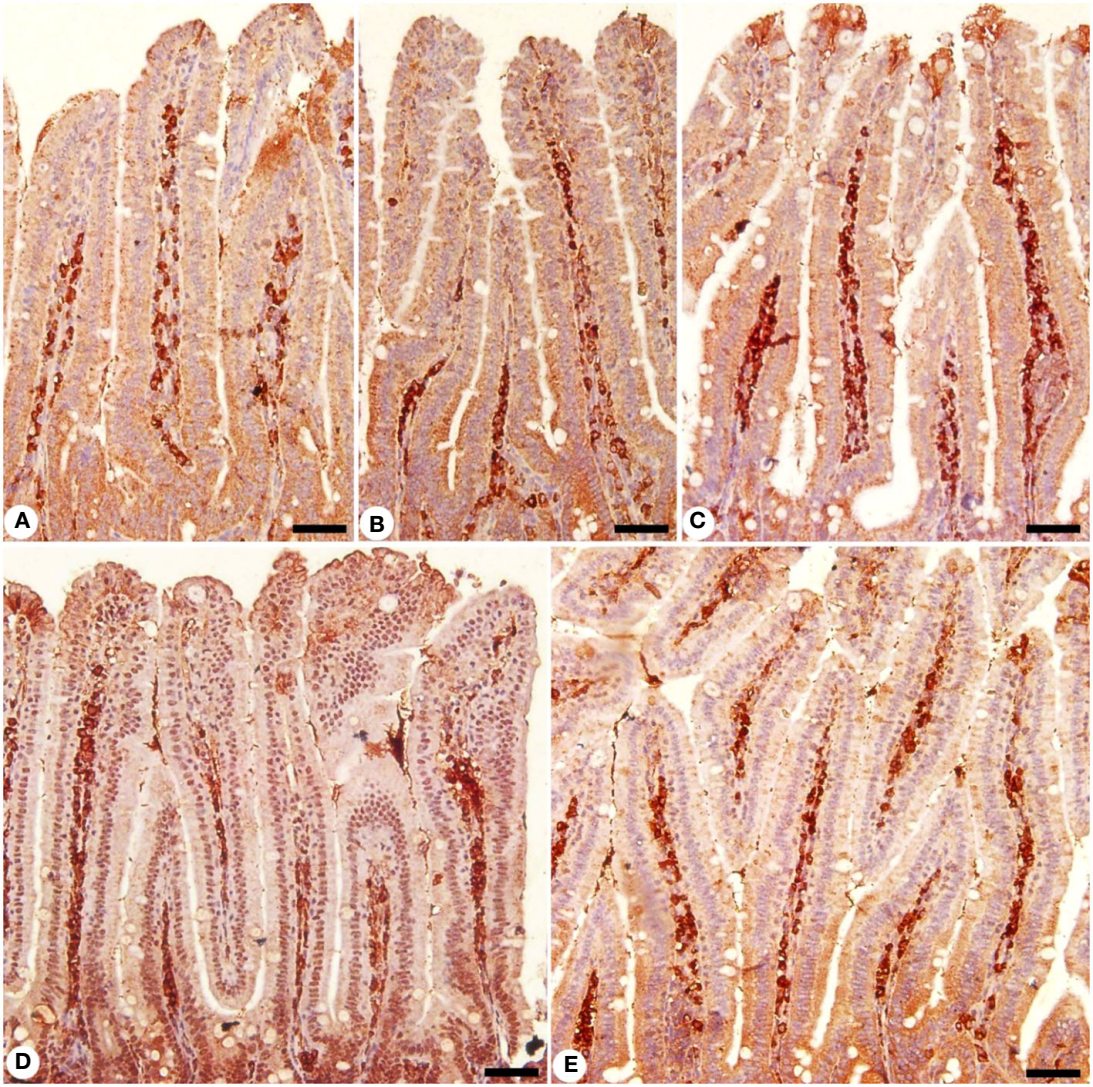
Immunohistochemical localization of NF-KB in the jejuna of mice. **(A)** control non-infected jejunum. **(B)** non-infected-treated group with 200 mg/kg KLRE. **(C)** *E. papillata* infected jejunum with an increased number of NF-KB-positive cells. **(D, E)** infected treated mouse (200 mg/kg KLRE and 120 mg/kg AMP, respectively) with a decreased number of NF-KB positive cells. Scale bar = 50µm.

**Figure 11:**
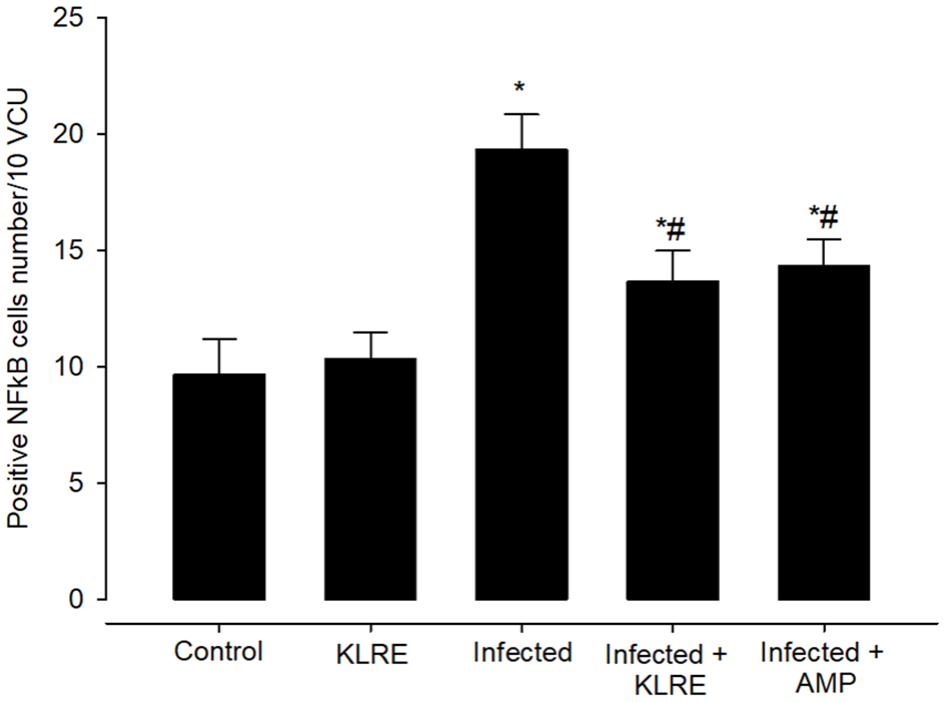
Positive NFkB cells number in mice infected with *Eimeria papillata* and for infected treated groups with 200 mg/kg KLRE and 120 mg/kg AMP. ^*^ significant change concerning the control group, ^#^ significance change concerning the infected group.

Moreover, jejunal sections were stained for NF-KB expression which play an important role in the inflammation and immune response caused by *Eimeria* infection (**Figures 10**, **11**). It showed that the *Eimeria* infection induced an elevation of the NF-KB expression level with a number of positive cells reached 193.31 ± 15.02 in the infected group compared to the normal status in the control group 96.63 ± 15.20 (**Figure 11**). Upon treatment, the expression of NF-KB significantly changed in contrast to the infected group to be 136.54 ± 13.22 in the infected-treated KLRE group and 143.65 ± 11.17 in the infected-treated AMP group (**Figures 10**, **11**).

**Figure 12:**
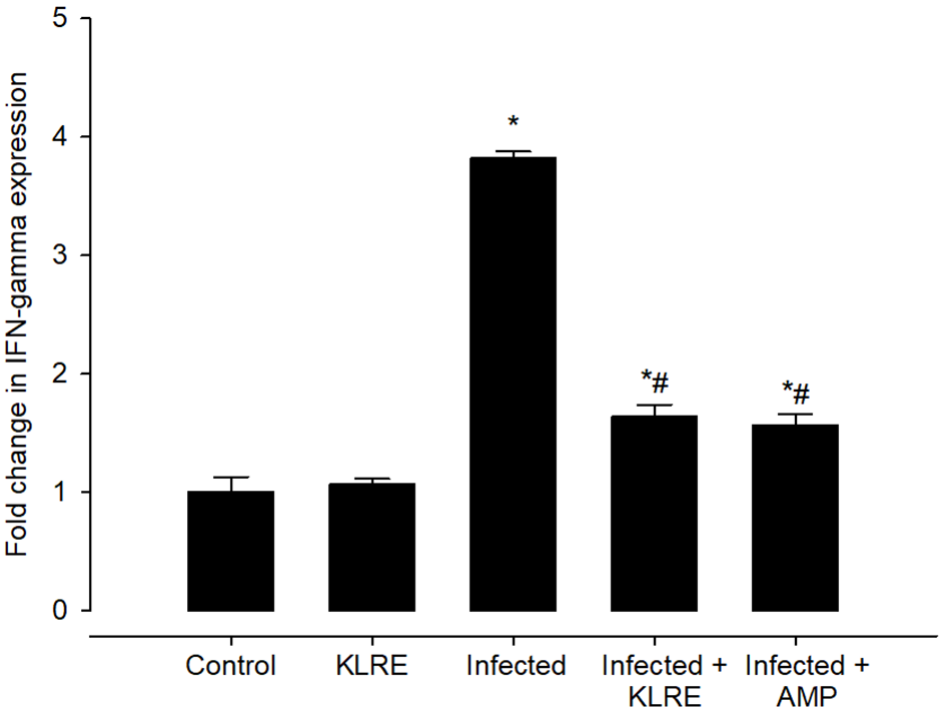
Effect of KLRE on the mRNA expression of *IFN- γ* in the jejunal samples from *E. papillata*-infected mice. The expression values obtained by RT-PCR analysis were normalized to the reference gene B-actin mRNA level and are shown as fold induction (in log 2 scale) relative to the mRNA level in the control. ^*^ significant change concerning the control group, ^#^ significance change concerning the infected group.

**Figure 13:**
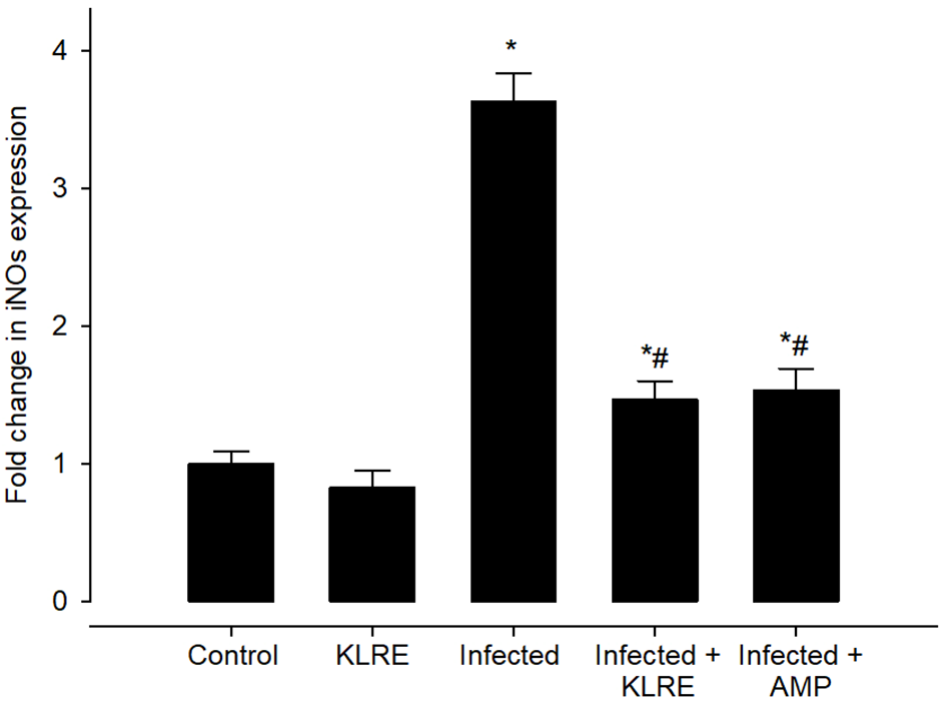
Effect of KLRE on the mRNA expression of *iNOs* in the jejunal samples from *E. papillata*-infected mice. The expression values obtained by RT-PCR analysis were normalized to the reference gene B-actin mRNA level and are shown as fold induction (in log 2 scale) relative to the mRNA level in the control. ^*^ significant change concerning the control group, ^#^ significance change concerning the infected group.

*Eimeria* infection increases mRNA expression of the *IFN-γ* gene in the jejunal tissue by around 3.81-fold compared to the control group (**Figure 12**). KLRE treatment significantly reduced *IFN-γ* gene expression by 1.63 fold, surpassing the reference drug’s 1.56 fold (**Figure 12**). Moreover, qRT-PCR demonstrated that *E. papillata* infection caused an increase in the mRNA expression level of the *iNOs* gene in the mice jejunum (**Figure 13**). Spontaneous enhanced nitric oxide (NO) production is linked to abnormal *iNOs* expression during *Eimeria* infection. After treatment with KLRE, *iNOs* gene expression was drastically reduced from 3.63 to 1.46 fold (**Figure 13**).

**Figure 14:**
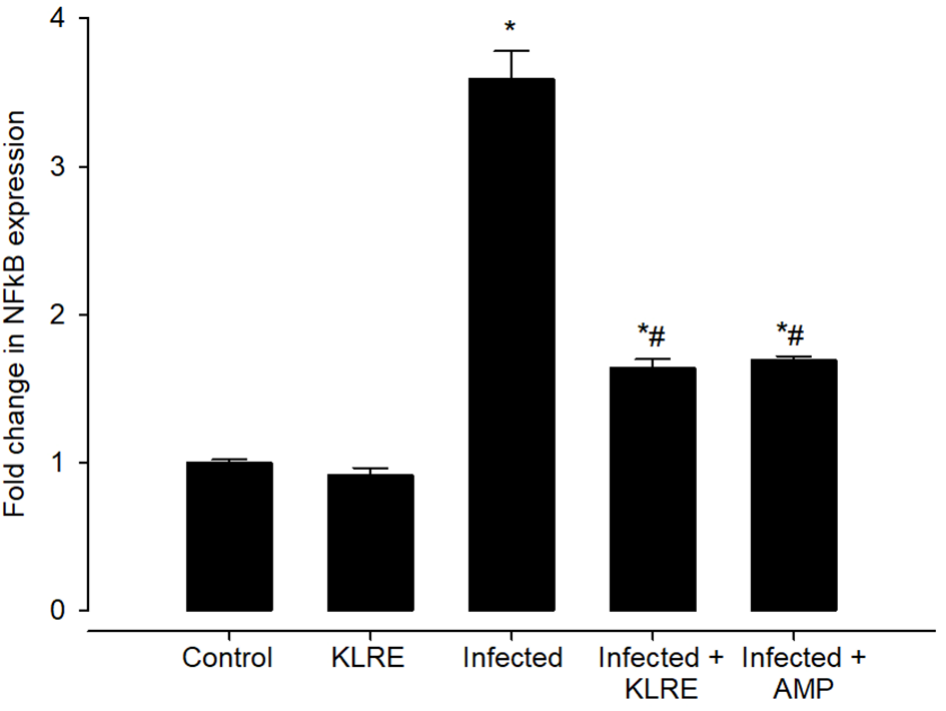
Effect of KLRE on the mRNA expression of *NF-kB* in the jejunal samples from *E. papillata*-infected mice. The expression values obtained by RT-PCR analysis were normalized to the reference gene β-actin mRNA level and are shown as fold induction (in log 2 scale) relative to the mRNA level in the control. ^*^ significant change concerning the control group, ^#^ significance change concerning the infected group.

The upregulation in the mRNA expression of the *NFkB* gene was observed after the *E. papillata* infection. This elevation in mRNA expression of this gene was about 3.59 fold when compared with the control group (1.00 fold) (**Figure 14**). *NFkB* promotes T helper 1 cell differentiation by regulating toll-cell receptor (TCR) signaling as well as functioning in innate immune cells to mediate induction of cytokines. Upon treatment with KLRE, a significant downregulation to about 1.64 fold was observed for the expression of the *NFkB* gene which is quite similar to the reference drug (1.69 fold) (**Figure 14**).

**Figure 15:**
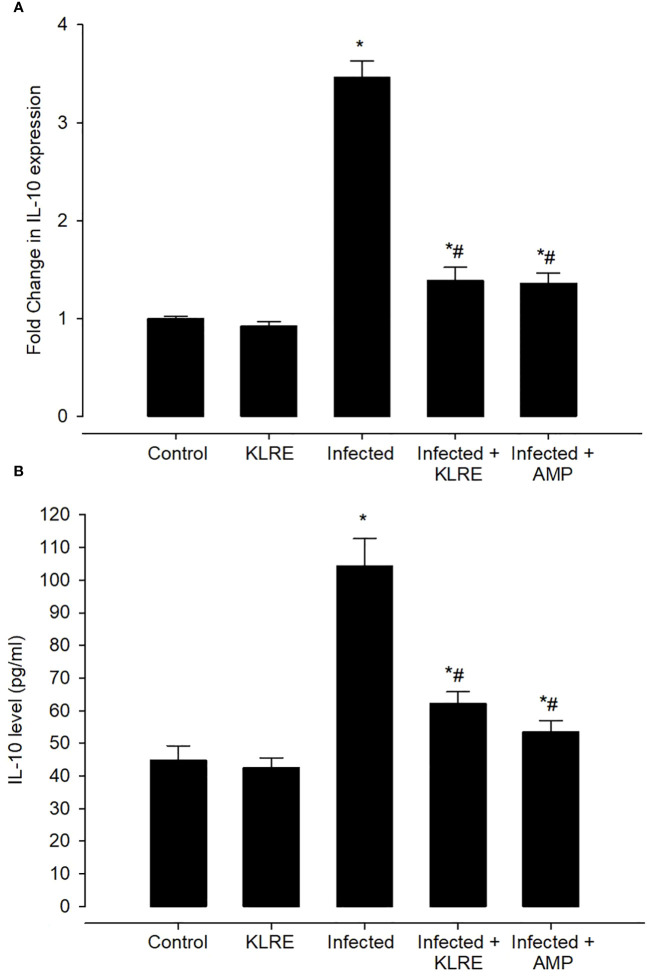
The effect of KLRE on IL-10 levels in *E. papillata*-infected jejunum. Gene expression results are presented as mean ± SD from triplicate assays, normalized to GAPDH, while biochemical assay results are displayed as mean ± SD values (n = 5). Panels **A**, **B** showcase IL-10 expression and levels. ^*^ denotes significance against the control group at P < 0.05, ^#^ denotes significance compared to the sepsis group at P < 0.05.

qRT-PCR was performed to assess changes in mRNA expression levels for inflammatory cytokines in the mice jejunum (**Figure 15**). *E. papillata* infection causes an increase in the mRNA expression of the *IL-10* gene after the immune activation of lymphocytes and macrophages. This gene’s mRNA expression increased by approximately 3.46-fold when compared to the control group (**Figure 15**). *IL-10* is a major anti-inflammatory mediator that protects mice against overactive reactions to *E. papillata*. Treatment with KLRE resulted in a significant downregulation of this gene’s expression by around 1.38 fold, which is similar to the reference drug (1.36 fold) (**Figure 15**). Moreover, analysis of IL-10 protein expression in the mice jejunum by ELISA revealed an increase in IL-10 production after infection with *E. papillata* reaching 104.27 ± 8.41 pg/ml, whereas KLRE treatment had significantly decreased IL-10 protein level (62.18 ± 3.63 pg/ml) at the 5^th^-day p.i. compared to the infected group (**Figure 15**).

## Discussion

Coccidiosis is typically treated with synthetic anticoccidial drugs; however, this strategy is under threat from the development of resistance in *Eimeria* strains (36). Different alternatives and techniques were successfully employed worldwide to treat and control diverse animal species (37). Among these options, numerous compounds derived from natural medicinal plants or natural health alternatives have been shown to have therapeutic effects that not only target the parasite but also preserve the host’s organs (38). This study sought to assess the anticoccidial and antioxidant properties as well as immunomodulation of *K. lappacea* roots against *Eimeria* infection. Previous studies reported the effective role of other root extracts like *Cassia sieberiana* (39), *Beta vulgaris* (40), *Salvadora persica* (41), and *Glycyrrhiza glabra* (42).

In addition to having a diminishing effect on the intracellular stages of the *Eimeria* parasite in the mice jejunum, the KLRE was an effective agent in mitigating infection, reducing oocyst shedding rate by approximately 75.71% on the 5^th^-day p.i. The majority of anticoccidial drugs have been shown to suppress *Eimeria* infection. *K. lappacea* derives its anticoccidial activity from many pharmacological compounds including antioxidants and phenolic compounds in its roots, which is consistent with Baumgartner et al. (14) Al-Oqail (18), and Alamari et al. (43). These compounds have a strong antimicrobial effect by disrupting the cell membrane of microbial pathogens leading to impaired membrane functions and leakage of cellular constituents and finally to cell death (19–22, 44). Most anticoccidial drugs have been shown to impede and inhibit the intracellular development of *Eimeria* stages in the intestinal tract, which is consistent with our findings.

The primary mechanism affected by *Eimeria* infection is host cell metabolism, and the parasite is very capable of manipulating host cells to its benefit by scavenging available host nutrients (45). Our data revealed that both carbohydrate and protein levels have been altered in the mice jejuna. Metwaly et al. (46) suggest that the lowered carbohydrate levels are due to the *Eimeria* stages’ excessive consumption of the stored carbohydrate content in the epithelial cells of mice jejuna. Regarding the bioactive components of KLRE, which disrupts the parasite’s ability to feed by lowering the enzymatic activity of glucose-6-phosphatase and thus plays an important role in the homeostatic regulation of tissue glycogen level, KLRE restored jejunal carbohydrate content. According to Al-Quraishy et al. (47), protein-losing enteropathy is characterized by the shedding of high amounts of proteins in the mice jejuna because of *Eimeria* infection. According to Bangoura and Daugschies (48), the amount of proteins in infected jejunal tissues is reduced, which is related to a higher rate of protein escaping into the intestinal lumen through the ruptured intestinal wall and being expelled via fecal pellets. The jejunal protein level improved after KLRE treatment due to a reduction in tissue protein catalytic processes.

The coccidian infection causes an imbalance in endogenous antioxidant defense and free radical production (49). Our findings showed that *Eimeria* infection induces oxidative damage to the mice’s jejunum, depleting antioxidant enzymes and lowering CAT and SOD levels. These oxidative indicators play an important role in protecting the animal body from free radical damage due to increased accumulation of reactive oxygen species (ROS) during *Eimeria* infection. Previous research has found that *Eimeria* infection disrupts the antioxidant defense system, resulting in detrimental cellular effects (10, 50–54). In this study, KLRE significantly prevented *Eimeria* infection-induced loss of these markers (CAT and SOD) and increased their activity, which is consistent with Carini et al. (55) for free radical scavenging properties that protect against oxidative damage and get rid of excess peroxides.

Furthermore, the elevated MDA and NO levels in infected mice are most likely caused by oxidative stress during *Eimeria* infection. Similar to the findings of Al-Otaibi et al. (45) Dominquez et al. (56)Al-Quraishy et al. (57), and Abdel-Gaber et al. (5) that *Eimeria*’s infective sporozoite stages, causing free radical overproduction and increased ROS production, resulting in lipid peroxidation. ROS causes pro-inflammatory cytokines and chemokines to be released, either directly or indirectly promoting inflammation due to *Eimeria* infection. In this study, *E. papillata* infection induced oxidative stress by upregulating the mRNA expression of *iNOs*, which is consistent with Metwaly et al. (58)Abdel-Latif et al. (59), and Abdel-Tawab et al. (30) reported that macrophages produce NO by oxidizing the guanidino nitrogen of L-arginine by an enzyme, nitric oxide synthase (NOS). This enzyme is inducible in macrophages by pathogen endotoxins and is termed inducible NOS (iNOS). The elevation of NO observed in this study is consistent with Allen and Fetterer (60) who stated that NO is involved in immunity and resistance against infectious diseases, as it exhibits toxicity towards pathogens. Administration of KLRE to the infected mice could significantly reduce the severity of the infection in the mice’s jejunum. Our findings showed that KLRE does not only target *Eimeria* stages within infected tissue but also exhibits anti-inflammatory activity protecting host tissues. The antioxidative, anti-inhibitory, and anti-inflammatory properties of KLRE may be attributed to the presence of phenolic and flavonoid components which mitigate the adverse effects of *Eimeria* infection on biological parameters of the affected animal. This is consistent with Awaad et al. (61) hypothesis that antioxidant compounds in different plants have played an important role in increasing the protection level against coccidiosis.

To understand the interaction between *Eimeria* infection and mice’s immune response, this study includes the determination of mRNA transcription levels for markers of T helper cell response. Lillehoj and Lillehoj (62) reported that T cell-mediated immunity by intestinal intraepithelial lymphocytes, including T helper (CD4) and T cytotoxic (CD8), confers the main component of protective immunity to *Eimeria* infections. This is consistent with the findings of this study that there is a significantly higher number of CD4 cells were detected in the jejunal tissue of infected mice compared to the control group, indicating macrophage involvement which migrates from circulation to the site of infection for the destruction of *Eimeria* stages. Previous studies by Dimier et al. (63) and Dalloul et al. (64) demonstrated that macrophages massively infiltrate into the chicken cecal lamina propria after *E. tenella* infection and secrete large amounts of cytokines. Generally, cytokines such as IL-1β, IL-12, IFN-γ, and TNF-α promote the development of cellular-mediated immunity against intracellular infections including coccidiosis (65). In addition, Mussbacher et al. (66) reported that NFkB may involve the expression of various pro-inflammatory genes, encoding cytokines and chemokines, and cell survival. This group of cytokines is associated with inflammatory responses, whilst cytokines such as IL-10 favor the development of humoral-mediated immunity and are implicated in anti-inflammatory responses (59, 67). This study showed that for mice inoculated with *E. papillata* oocysts, *IL-10*, *NFkB*, and *IFN-γ* during the 5^th^-day p.i., their gene expression levels were fold increased which in turn produces reactive oxygen (ROS) leading to the upregulated NO and iNOs levels, consistent with oocysts shedding compared with the control group. This is consistent with Lyons et al. (68) reported that a single infection with *E. tenella* evoked a host immune response which led to significant expression of cytokines such as *IL-10* and *IFN-γ* in the ceca. Similar to the findings of Hong et al. (69) *IL-10* and *IFN-γ* mRNA expression was robustly increased in CD4 cell populations following *E. maxima*-infected chickens. Our findings also indicated that the ELISA assay also detected massive IL-10 protein expression positively correlated with *E. papillata* replication and it is plausible *E. papillata* is inducing IL-10 as an immune evasion strategy. This indicated that T cells mediate their effects on the *Eimeria* parasite in primary infections through the secretion of cytokines. Similar to the findings of Bremner et al. (70) reported the increased IL-10 level in serum after *E. maxima* infection. Moreover, Chow et al. (71) and Dkhil et al. (72) reported that *NFkB* and *IFN-γ* is a key cytokine orchestrating the development of cellular-mediated immunity, and its expression is regulated by the induction of *IL-10*. It is believed that IFN-γ, which is known to activate intracellular cytotoxicity and produced by natural killer T cells, stimulates neutrophils and macrophages to migrate from circulation to the site of infection to destroy *Eimeria* sporozoites. This is consistent with previous studies demonstrated that a strong IFN-γ has been described to occur in the intestine upon infection with *E. maxima* (73), *E. bovis* and *E. alabamensis* (74). Our findings indicated that KLRE protects host tissues by acting as an immunomodulatory agent in addition to targeting intracellular *Eimeria* stages within the infected jejunal tissues associated with inhibiting fecal oocyst shedding as well as impairing the development and maturation of parasites. Following this view, our findings that KLRE attenuates the inflammatory response since it significantly downregulates the mRNA expression of *IL-10*, *NFkB*, and *IFN-γ* in the mouse jejunum infected with *E. papillata*. This reduction in *iNOs, IL-10, NFkB*, and *IFN-γ* in the intestinal tissue confirms the immunomodulatory and anti-inflammatory properties of flavonoid compounds in KLRE as well as their capacity to reduce the production of cytokines, which agreed with previous studies of Femández et al. (75), and Baumgartner et al. (14).

## Conclusion

Our findings collectively demonstrate that the root extract of *Krameria lappacea* possesses anticoccidial properties, along with a notable enhancement in the nutritional status of jejunal tissue. Moreover, it exhibits antioxidant and anti-inflammatory activities, safeguarding host tissues from injuries induced by *E. papillata*. Further investigations are warranted to explore its potential protective role in other organs, as well as to conduct biochemical and molecular analyses to identify the genes regulated during infection.

## Data availability statement

The raw data supporting the conclusions of this article will be made available by the authors, without undue reservation.

## Ethics statement

This research was approved by the Research Ethics Committee (REC) at King Saud University (approval number KSU-SU-23-127). The study was conducted in accordance with the local legislation and institutional requirements.

## Author contributions

RA-G: Conceptualization, Data curation, Formal analysis, Funding acquisition, Investigation, Methodology, Project administration, Resources, Software, Supervision, Validation, Visualization, Writing – original draft, Writing – review & editing. GA: Formal analysis, Investigation, Methodology, Visualization, Writing – original draft, Conceptualization, Data curation, Funding acquisition, Project administration, Resources, Software, Supervision, Validation, Writing – review & editing. MD: Formal analysis, Investigation, Methodology, Project administration, Software, Visualization, Writing – review & editing, Conceptualization, Data curation, Funding acquisition, Resources, Supervision, Validation, Writing – original draft. AM: Investigation, Validation, Visualization, Writing – review & editing, Conceptualization, Data curation, Formal analysis, Funding acquisition, Methodology, Project administration, Resources, Software, Supervision, Writing – original draft. EA-S: Data curation, Methodology, Resources, Validation, Visualization, Writing – review & editing, Conceptualization, Formal analysis, Funding acquisition, Investigation, Project administration, Software, Supervision, Writing – original draft. SA-Q: Investigation, Methodology, Project administration, Resources, Supervision, Validation, Visualization, Writing – review & editing, Conceptualization, Data curation, Formal analysis, Funding acquisition, Software, Writing – original draft.
